# Establishment of a malignancy and benignancy prediction model of sub-centimeter pulmonary ground-glass nodules based on the inflammation-cancer transformation theory

**DOI:** 10.3389/fmed.2022.1007589

**Published:** 2022-10-05

**Authors:** Changxing Shen, Qiong Wu, Qing Xia, Chuanwu Cao, Fei Wang, Zhuang Li, Lihong Fan

**Affiliations:** ^1^Department of Integrated Traditional Chinese and Western Medicine, Shanghai Tenth People’s Hospital, Tongji University School of Medicine, Shanghai, China; ^2^Liangcheng Xincun Community Health Services Center, Shanghai University of Traditional Chinese Medicine, Shanghai, China

**Keywords:** prediction model, sub-centimeter, pulmonary GGNs, Mayo model, innovative

## Abstract

**Background:**

In recent years, Chinese clinicians are frequently encountered by patients with multiple lung nodules and these intensity ground-glass nodules (GGNs) are usually small in size and some of them have no spicule sign. In addition, early lung cancer is diagnosed in large numbers of non-heavy smokers and individuals with no caner history. Obviously, the Mayo model is not applicable to these patients. The aim of the present study is to develop a new and more applicable model that can predict malignancy or benignancy of pulmonary GGNs based on the inflammation-cancer transformation theory.

**Materials and methods:**

Included in this study were patients who underwent surgical resection or lung puncture biopsy of GGNs in Shanghai 10th People’s Hospital between January 1, 2018 and May 31, 2021 with the inclusion criterion of the maximum diameter of GGN < 1.0 cm. All the included patients had their pulmonary GGNs diagnosed by postoperative pathology. The patient data were analyzed to establish a prediction model and the predictive value of the model was verified.

**Results:**

Altogether 100 GGN patients who met the inclusion criteria were included for analysis. Based on the results of logistic stepwise regression analysis, a mathematical predication equation was established to calculate the malignancy probability as follows: Malignancy probability rate (p) = ex/(1 + ex); *p* > 0.5 was considered as malignant and *p* ≤ 0.5 as benign, where *x* = 0.9650 + [0.1791 × T helper (Th) cell] + [0.2921 × mixed GGN (mGGN)] + (0.4909 × vascular convergence sign) + (0.1058 × chronic inflammation). According to this prediction model, the positive prediction rate was 73.3% and the negative prediction rate was 100% versus the positive prediction rate of 0% for the Mayo model.

**Conclusion:**

By focusing on four major factors (chronic inflammation history, human Th cell, imaging vascular convergence sign and mGGNs), the present prediction model greatly improves the accuracy of malignancy or benignancy prediction of sub-centimeter pulmonary GGNs. This is a breakthrough innovation in this field.

## Background

Lung cancer is a malignant tumor ranking first in terms of both the incidence and mortality worldwide, primarily because of lacking early prediction and intervention. The Mayo model is a classic model for malignancy probability prediction of pulmonary nodules *via* synthetic analysis of age, smoking history, extra-thoracic tumor history, nodule diameter, the presence or absence of the spicule sign, and whether or not the nodule is located in the upper lung lobe. However, the pulmonary ground-glass nodules (GGNs) encountered in clinical practice are mostly multi-focal and small in size with no spicule sign. Furthermore, early lung cancer is diagnosed in large numbers of non-heavy smokers and individuals with no caner history (1, 2). In addition, more studies have reported chronic inflammation-cancer transformation in more sub-healthy individuals with pulmonary GGNs complicated with organ and tissue chronic inflammation due to the influence from the natural environment, food safety, and psychic pressure (3–5). For this reason, we need to ponder over the impact of these previously ignored factors on the pathogenesis of lung cancer and the predictive value, and establish a new and more applicable model for predicting malignancy or benignancy of pulmonary nodules for the sake of improving the diagnostic accuracy of early lung cancer, especially in China.

## Patients and methods

Included in this study were patients who underwent surgical resection of GGNs in Shanghai 10th People’s Hospital between January 1, 2018 and May 31, 2021 with the inclusion inclusion criterion of the maximum diameter of GGN < 1.0 cm.

The inclusion criteria were (1) patients who were found to have solitary nodules in the lung parenchyma on the CT image of our hospital; (2) the diameter of the nodule was 5 mm < diameter < 10 mm; (3) complete case information was available; (4) the diagnosis was confirmed by postoperative pathology; (5) tumor markers of CEA, SCC, NSE, and CYFRA21-1 were available; (6) all pulmonary nodules were followed up for more than 3 months; and (7) the patients had not received any preoperative treatment.

The exclusion criteria were (1) patients with diffuse pulmonary nodules; (2) solid pulmonary nodules; (3) calcified pulmonary nodules; (4) clinical data were incomplete; (5) patients who did not receive CT examination in our hospital; and (6) patients who had received chemotherapy, radiotherapy, or other treatments before operative.

### Clinical data collection

Clinical data were collected from patients who had found pulmonary nodules in physical examinations but without receiving any treatment, including the sex, age, smoking history, personal tumor history, family tumor history, inflammation history of other tissue, and organs or chronic pulmonary inflammation history, and anxiety (international standard anxiety self rating scale, SAS) or depression (Self rating Depression Scale, SDS).

### Histopathological criteria for judging pulmonary nodules

Malignancy of pulmonary nodules was confirmed by postoperative pathology of the surgical resected specimens. Benignancy of pulmonary nodules was confirmed by postoperative pathology of the surgical resected specimens. 100 patients underwent segmental pneumonectomy, and 100 nodule specimens were removed.

### Laboratory evaluation

Blood inflammatory cytokines, T cell subsets and tumor biomarkers were detected within 1 month before surgery.

### Imaging information collection

Imaging information included the pulmonary nodule type [mixed GGN (mGGN), pure GGN (pGGN)], whether the nodular lesion was accompanied with the vascular convergence sign, the spicule sign, a smooth or unsmooth boundary, the presence or absence of pleural indentation, the presence or absence of the vacuole sign, nodule diameter (mm), and whether or not the nodule was located in the upper lung lobe.

### Statistical methods

The nodules were grouped according to the pathological results. Comparison between two groups was statistically analyzed by R language. The above 19 risk factors were analyzed by univariate analysis (whether the laboratory indexes were elevated or not, the baseline medical history, personal history and family history, and imaging signs were defined as binary variables: yes, 1, and no, 0; elevated, 1, and normal, 0). Significant differences in univariate analysis were subjected to multivariate logistic regression analysis. *p* < 0.05 was defined as statistically significant. Significant risk factors were used to establish a mathematical prediction equation as follows: Malignancy probability rate (p) = ex/(1 + ex); *p* > 0.5 was considered as malignant and *p* ≤ 0.5 as benign.

## Results

In the model group, 60 patients were diagnosed as having malignant pulmonary nodules by postoperative pathology of the surgically resected specimens, and 60 nodular lesions were treated; 40 patients were diagnosed as having benign pulmonary nodules by postoperative pathology of the surgically resected specimens, and 40 lesions were treated (Table 1). The results of univariate analysis of the 19 risk factors were shown in Table 2, compared with patients with benign pulmonary nodules, the results of differential analysis suggested that patients with malignant pulmonary nodules had significant differences in serum inflammatory factors, immune cells, imaging characteristics, history of chronic inflammation, history of tumor, and psychological factors (Table 2). Then Multivariate logistic stepwise regression analysis indicated that Th (helper T) cell reduction, mGGN, vascular convergence sign and chronic inflammatory disease history were significantly correlated with malignant nodules (Table 3). Based on the results of logistic stepwise regression analysis, a mathematical predication equation was established to calculate the malignancy probability as follows: Malignancy probability rate (*p*) = ex/(1 + ex); *p* > 0.5 was considered as malignant and *p* ≤ 0.5 as benign. **X = 0.9650 + (0.1791 × Th cell) + (0.2921 × mGGN) + (0.4909 × vascular convergence sign) + (0.1058 × chronic inflammation)**

**TABLE 1:** Pathological results of pulmonary nodules in the model group.

Pathological type	Pathological result	*n* = 100
**Malignant**		***n* = 60**
	*In situ* cancer	30
	Adenocarcinoma	24
	Squamous carcinoma	3
	Small-cell lung cancer	1
	Other malignant types	2
**Benign**		***n* = 40**
	Inflammatory granuloma	18
	Tuberculoma	8
	Hamartoma	5
	Hemangioma	4
	Inflammatory pseudotumor	3
	Others	2

**TABLE 2:** Comparison of risk factors between malignant and benign groups in the model group.

Factors	Malignant group *n* = 60	Benign group *n* = 40	*P*
Sex (M/F)	27/33	20/20	0.09
Age (yr.)	51.82 ± 14.84	52.48 ± 10.40	0.12
Proinflammatory factor elevation (yes/no)	42/18	12/28	<0.05*
Th cell reduction (yes/no)	54/6	10/30	<0.05*
Tumor marker elevation (yes/no)	18/42	7/33	0.07
Mixed GGNs (yes/no)	56/4	5/35	<0.05*
Pure GGNs (yes/no)	2/58	10/30	<0.05*
Vascular convergence sign (yes/no)	60/0	6/34	<0.05*
Short spicule sign (yes/no)	4/56	0/40	0.56
Smooth boundary (yes/no)	0/60	10/30	<0.05*
Pleural indentation (yes/no)	8/52	0/40	0.19
Vacuole sign (yes/no)	6/54	2/38	0.21
Nodule diameter (mm)	9.43 ± 2.21	10.75 ± 2.42	0.18
Nodule in upper lobe (yes/no)	17/43	4/36	<0.05*
Smoking history (yes/no)	9/51	5/35	0.77
Personal tumor history (yes/no)	12/48	2/38	<0.05*
Family tumor history (yes/no)	4/56	1/39	<0.05*
Chronic inflammatory disease history (yes/no)	20/40	4/36	<0.05*
Anxiety/depression history (yes/no)	13/47	2/38	<0.05*

Th cell, T helper cell. *Statistically significant.

**TABLE 3:** Logistic regression analysis of risk factors for malignant nodules in the model group.

Factors	*B*	S.E.	*P*
Proinflammatory factor elevation (yes/no)	0.0047	0.0439	0.21
Th cell reduction (yeas/no)	0.1791	0.0524	0.01*
mGGNs (yes/no)	0.2920	0.0664	0.001*
pGGNs (yes/no)	–0.1061	0.0679	0.12
Vascular convergence sign (yes/no)	0.4908	0.0695	0.02*
Smooth boundary (yes/no)	–0.1199	0.0725	0.22
Nodule in upper lobe (yes/no)	0.0677	0.0516	0.15
Personal tumor history (yes/no)	0.0024	0.0590	0.41
Family tumor history (yes/no)	0.1289	0.0825	0.32
Chronic inflammatory disease history (yes/no)	0.105845	0.046626	0.01*
Anxiety/depression (yes/no)	0.0034	0.0027	0.09

*Statistically significant.

where *e* is a natural logarithm:

if the patient’s Th cell was reduced before operation, Th cell = 1 (otherwise = 0);

if the patient’s pulmonary nodule was mGGN before operation, mGGN = 1 (otherwise = 0);

if the patent’s pulmonary nodule presented vascular convergent sign before operation, vascular convergent sign = 1 (otherwise = 0);

if the patient was complicated with a chronic inflammatory disease history, chronic inflammation = 1 (otherwise = 0).

**FIGURE 1:**
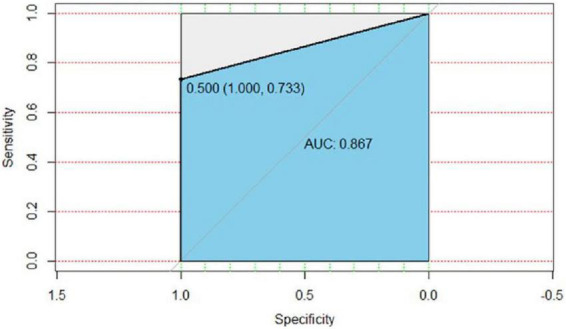
ROC curve of the validation group of the present model. AUC = 0.867, the specificity was 100%, the sensitivity was 73.3%, and the cut-off value of logistic regression was 0.5.

In the validation group, 27 patients were diagnosed as having malignant pulmonary nodules by postoperative pathology of the surgically resected specimens or puncture biopsy, and 22 nodular lesions were treated; 18 patients were diagnosed as having benign pulmonary nodules by postoperative pathology of the surgically resected specimens, and 18 lesions were treated (Table 4). The predictive efficacy of the mathematical prediction model was judged by entering the data of the validation group into the model of the present study, using the pathological results as the gold standard, and drawing ROC curves by R language statistical software, AUC = 0.867. As shown in Figure 1, the positive prediction rate of our prediction model was 73.3%, and the negative prediction rate was 100% (Table 5). Compared with the new prediction model, Mayo model equation: *X* = −6.8272 + (0.0391 × age) + (0.7917 × smoking history) + (1.3388 × malignant tumor history) + (0.1274 × diameter) + (1.0407 × spicule) + (0.7838 × upper lobe). When the validation model data were entered into the Mayo model, the positive prediction value was 0 (Table 6).

**TABLE 4:** Baseline data of patients in malignant and benign groups of the validation group.

Factors	Malignant group *n* = 22	Benign group *n* = 18	*p*
Sex (M/F)	12/10	9/9	0.86
Age (yr.)	54.55 ± 12.09	50.50 ± 8.43	0.09

**TABLE 5:** Results of the present prediction model.

Pathological gold standard	Model prediction diagnosis		
	
	Malignant	Benign	Total
Malignant	22	8	30
Benign	0	10	10
Total	22	18	40

**TABLE 6:** The Mayo model prediction result.

Pathological gold standard	Model prediction diagnosis		
	
	Malignant	Benign	Total
Malignant	0	0	0
Benign	22	18	40
Total	22	18	40

## Discussion

The incidence of sub-centimeter pulmonary nodules is high in China. As most of these nodules grow slowly and the volume doubling time varies from individual to individual, the diagnostic value of long-term follow-up observation of imaging changes of the nodules is often limited and may cause misdiagnosis of cancer (6). In the present study, we found that the positive prediction rate of the Mayo model on sub-centimeter pulmonary nodules was very low and not suitable for predicting the nature of sub-centimeter pulmonary nodules. In recent years, various models for predicting the nature of pulmonary nodules have been reported. Most of them are based on different pathophysiological characteristics of pulmonary nodules, including the prediction model by combining ctDNA with the imaging features, the prediction model based on pulmonary bloodstream transmission time, the prediction model using human plasma protein LG3BP and C163A, the prediction model using serum miRNA-21-5p, and imaging prediction model (7–15). Sub-centimeter GGNs are mostly *in situ* cancer with relatively low activity of cancer cell metabolism. For this reason, the diagnostic reliability of PETCT-based prediction model is questionable. In addition, most sub-centimeter pulmonary nodules have no short spicule sign, and the incidence of sub-centimeter pulmonary nodules does not increase with the increasing age of the patients (16, 17). As most of these prediction models only use a single aspect of risk factors to assess malignancy or benignancy of the nodules, their clinical prediction value is limited. To the best of our knowledge, there is no model that simultaneously uses a comprehensive spectrum of multiple factors to predict the nature of pulmonary GGNs with relatively small volumes.

Patients with pulmonary nodules are likely to have varying degrees of anxiety and depression, leading to immune dysfunction and low-grade inflammation (18). The inflammation-cancer transformation mechanism has been generally accepted, knowing that long-term chronic inflammation can lead to alterations of the human homeostasis, such as elevation of interleukin-6 (IL-6) as the representative of proinflammatory cytokines and decrease of Th cells in the body. In addition, tissue cell proliferation occurs upon inflammatory stimulation, which promotes new vessel formation to ensure nutrient supply (19). Chronic inflammation-mediated immune response change is an important regulatory factor for the development and evolution of human diseases (20). Studies have demonstrated that a high level of circulating IL-6 is correlated with adverse prognosis of various cancer types including non-small cell lung cancer (NSCLC). Inflammatory cytokines can stimulate the immune system and promote tumor growth, and change of T cell group and function is probably one of the mechanisms underlying the adverse prognosis of NSCLC patients with elevated level of IL-6 (21). The increase of neutrophil compartments in circulation is related to the generation of progranulocytes (IL-1β, IL-17A, TNFα, and IL-6) and elevation of Th2-related cytokines. In tumor patients with a relatively high level of neutrophils, T cell immune response is often reduced, as represented by the reduced expression of cytotoxic T cell genes including CD8A, CD8B, GZMA, and GZMB, the reduced number of CD3 + CD8 + cells, and the reduced expression of IFN-γ related genes (22). Studies have demonstrated the level of inflammatory cytokines is significantly higher and the level of CD4/CD8 is significantly lower in lung cancer and GGN patients as compared with that in patients with benign nodules. In addition, the anti-inflammatory and immune functions are reduced in lung cancer and pulmonary GGN patients, and therefore inflammatory cytokines and immune function can also be used as references for lung cancer prediction (23). Nowadays, natural environmental pollution, food safety, exposure to various irradiations in daily life, pressures from various social aspects, psychic factors, and chronic persistent infections are all factors that can cause the human sub-healthy status. These factors work together to cause chronic inflammation in different parts of the body. Most recent data have expanded the concept about the key role that inflammation plays in cancer development and progression. It has become clearer that the tumor microenvironment (TME) is mainly coordinated by inflammatory cells, and inflammation is an indispensable participant of tumorigenesis by promoting cancer cell proliferation, survival and migration (24, 25). The data obtained in our study also support the chronic inflammation history and Th cell as the lung cancer prediction indexes.

Mixed GGNs are a high-risk factor of primary lung cancer which has been recognized by most clinicians and researchers, and micro-infiltrating lung adenocarcinoma is often manifested by mGGNs (26–28). Vascular convergence is closely correlated with lung adenocarcinoma (29, 30). Vascular lung cancer tumors with vascular convergence sign are often nourished by multiple blood vessels which get assembled in the lung cancer. The clinical significance of this sign indicates the rich blood supply of the nodular lesion and a high probability of malignancy. Some studies reported that the sensitivity, specificity, and accuracy of using the vascular convergence sign as an indicator of lung cancer was 97.2, 68.8, and 93.7%, respectively, which is helpful for lung cancer prediction (21–35). Starting from the pathogenesis of human pulmonary nodules, the prediction model described in this study made a comprehensive analysis of multiple factors that may affect the development and progression of lung cancer, including the chronic inflammation history, family tumor hereditary history, GGN imaging characteristic, serum level of inflammatory cytokines, and T cell subset indexes. The results verified by the validation group show that this prediction model can minimize the misdiagnosis of malignant lung nodules with a high prediction rate and therefore is worthy of clinical promotion.

## Conclusion

The prediction model described in this study is an innovative breakthrough in that it greatly improves the accuracy of predicting malignancy or benignancy of sub-centimeter pulmonary nodules by focusing on the four major factors (chronic inflammation history, human immune cell, imaging vascular convergence sign, and mGGNs), thus providing clinicians with an auxiliary tool for the diagnosis and interventional decision making of sub-centimeter pulmonary nodules.

## Data availability statement

All datasets generated for this study are included in the article/Supplementary material.

## Ethics statement

The studies involving human participants were reviewed and approved by the Ethics Committee of Shanghai Tenth People’s Hospital, approval number: SHSY-IEC-5.0/22K122/P01. The patients/participants provided their written informed consent to participate in this study.

## Author contributions

LF contributed substantially to the study design. QW and QX contributed substantially to the data providing. CS had full access to all data in the study, took responsibility for the integrity of the data, and the accuracy of the data analysis including and especially any adverse effects, and contributed substantially to the writing of the manuscript. CC and FW made contributions to the literature review, while ZL made contributions to the data processing. All authors contributed to the article and approved the submitted version.
