# Supplementary material for: Establishment of a malignancy and benignancy prediction model of sub-centimeter pulmonary ground-glass nodules based on the inflammation-cancer transformation theory
Source: Front Med (Lausanne). 2022 Oct 5;9:1007589. doi: 10.3389/fmed.2022.1007589 (PMC9581285; doi:10.3389/fmed.2022.1007589)
Supplement: Supplementary file 1 [file Data_Sheet_1.zip › SupMaterial/Editorial 1.PDF]

上海市第十人民医院伦理委员会审批件

声明：本伦理委员会按照国家卫计委和 CFDA 有关法规组成和工作，其审查和工作过程不受伦理委员会以外任何组织及个人的影响。

批件号：SHSY-IEC-5.0/22K122/P01

审查日期：	2022.5.28	本院伦理号：22K122
审查会议地点：	NA	
研究项目名称：	基于炎癌变理论的亚厘米肺磨玻璃结节的良恶性预测模型	
试验产品名称：	NA	
审查文件：	详见随附“上海市第十人民医院伦理委员会审查文件清单”	
申办者/CRO：	NA	
主要研究者：	范理宏	组长单位名称：NA
本院研究者：	范理宏	科室：中西医整合肺结节中心
主审委员：	徐亚伟 房林	
审查类别：	■初始审查 □复审 □修正案审查 □其他______	
伦理审查方式：	□会议审查 ■快速审查 □紧急会议审查	
审查结果：	1. ■同意 □修改后同意 □修改后重审 □不同意<br>2. 伦理委员会对该研究实施过程的年度/定期跟踪审查：■是 □否<br>审查频度为研究批准之日起： □3 个月 □6 个月 □9 个月 ■12 个月<br>3. 伦理委员会有权根据实际进展情况改变年度/定期跟踪审查频度。<br>4. 自批准之日起一年内项目未启动，该批件自动失效。	

主任或副主任委员签字：[signature]

日期：2022.5.29

上海市第十人民医院伦理委员会（盖章）

注意：（请仔细阅读）

1. 本伦理委员会批准的项目为涉及人体的生物医学研究，必须严格按照所批最新版本的研究方案和知情同意书开展研究，并遵循国内相关法规指南要求。
2. 凡是涉及人类遗传资源出口或者按照国家规定必须经有关部门专项审批的内容，均需在项目执行前向有关部门申报并获得批准。
3. 本批件可能用于其他中心伦理委员会参考，如果对方案审查存在不同意见，请及时与本伦理委员会沟通。
4. 对已批准的研究方案、知情同意书等材料的任何修改及主要研究者更换等，须及时通知本伦理委员会重新审查，获得批准后执行。
5. 发生严重不良事件及影响研究风险受益比的非预期事件，须及时报告本伦理委员会。
6. 根据伦理委员会对年度/定期跟踪审查频度的意见，无论研究开始与否，请在年度/定期跟踪审查日到期前 1 个月提出年度/定期跟踪审查的申请。
7. 发现不依从/违反方案情况须及时报告伦理委员会审查。
8. 暂停/提前终止临床研究，请及时通知伦理委员会。
9. 完成研究，须提交结题报告供伦理委员会审查。

地址：上海市静安区延长中路 301 号，电话：021-66301604
