# Supplementary material for: Establishment of a malignancy and benignancy prediction model of sub-centimeter pulmonary ground-glass nodules based on the inflammation-cancer transformation theory
Source: Front Med (Lausanne). 2022 Oct 5;9:1007589. doi: 10.3389/fmed.2022.1007589 (PMC9581285; doi:10.3389/fmed.2022.1007589)
Supplement: Supplementary file 1 [file Data_Sheet_1.zip › SupMaterial/Editorial 2.DOCX]

**Approval of the Ethics Committee of Shanghai Tenth People's Hospital**

Declaration: The Ethics Committee is composed and works in accordance with the relevant regulations of the National Health and Family Planning Commission and CFDA, and its review and work process are not influenced by any organization or individual outside the Ethics Committee.

Grant No.: SHSY-IEC-5.0/22K122/P01

| Date of review. | May 28, 2022 | | Internal hospital approval number | | 22K122 |
| --- | --- | --- | --- | --- | --- |
| Place of review meeting. | NA | | Name of the test product. | | NA |
| Name of research project. | Establishment of a malignancy and benignancy prediction model of sub-centimeter pulmonary GGNs based on the inflammation-cancer transformation theory | | | | |
| Reviewed documents. | Application form for Ethical Review, Research plan, Exemption of informed consent, Curriculum Vitae of principal investigator, Principal investigator statement. | | | | |
| Sponsor/CRO. | NA | | | | |
| Name of group leader. | NA | | | | |
| Principal Investigator. | NA | Investigator(s)/Department(s) in this institution. | | Lihong Fan / Integrated Traditional Chinese Medicine and Western Medicine Department | |
| Type of review. | ■ Initial review □ Review □ Amendment review □ Other | | | | |
| Ethics review method. | □ Meeting review ■ Expedited review □ Emergency meeting review | | | | |
| Lead reviewers. | Yawei Xu, Lin Fang | | | | |
| Review results. | 1. ■ Agree □ Agree after revision □ Re-examine after revision □ Disagree  2. Annual/regular follow-up review of the ethics committee on the implementation process of the study: ■Yes □No  The frequency of review is from the date of study approval: □ 3 months □ 6 months ■ 12 months  3. The Ethics Committee has the right to change the frequency of annual/periodic follow-up review according to the actual progress.  4. If the project is not initiated within one year from the date of approval, the approval will automatically expire. | | | | |
| Signature of the director or deputy director: Fenyong Sun  Ethics Committee of Shanghai Tenth People's Hospital (seal)  Date: May 29, 2022 | | | | | |
| Note: (Please read carefully)  1. The projects approved by this Ethics Committee are biomedical research involving human subjects and must be carried out in strict accordance with the latest version of the approved research protocol and informed consent form, and in compliance with the requirements of relevant domestic regulatory guidelines.  2. Any content that involves the export of human genetic resources or must be specifically approved by the relevant authorities according to national regulations must be declared to and approved by the relevant authorities prior to the implementation of the project.  3. This approval may be used for reference by the ethics committees of other centres. If there is a difference of opinion on the review of the protocol, please communicate with this ethics committee in a timely manner.  4. Any amendment to the approved research protocol, informed consent form and other materials, as well as change of principal investigator, etc., must be promptly notified to this Ethics Committee for re-examination and implementation after approval. | | | | | |
| 5. Serious adverse events and unintended events that affect the risk-benefit ratio of the research must be reported to the Ethics Committee in a timely manner.  6. Based on the Ethics Committee's opinion on the frequency of annual/periodic follow-up reviews, please apply for an annual/periodic follow-up review 1 month before the annual/periodic follow-up review date is due, regardless of whether the study has started or not.  7. Any non-compliance/violation of the protocol must be reported to the Ethics Committee for review in a timely manner.  8. Please notify the Ethics Committee in a timely manner of any suspension/early termination of the clinical study.  9. Upon completion of the study, a final report must be submitted for review by the Ethics Committee. | | | | | |

Address: No. 301, Yanchang Middle Road, Jing'an District, Shanghai, China, Tel: 021-66301604

SHSY-IEC-BG/05.08/04.1 Approval of the Ethics Committee of Shanghai Tenth People's Hospital
